# Integration of soil magnetometry and geochemistry for assessment of human health risk from metallurgical slag dumps

**DOI:** 10.1007/s11356-017-0218-5

**Published:** 2017-09-25

**Authors:** Marzena Rachwał, Małgorzata Wawer, Tadeusz Magiera, Eiliv Steinnes

**Affiliations:** 1 Institute of Environmental Engineering, Polish Academy of Sciences, 34 Sklodowska-Curie St, 41-819 Zabrze, Poland; 2 Department of Chemistry, Norwegian University of Science and Technology, Høgskoleringen 5, N-7491 Trondheim, Norway

**Keywords:** Soil magnetometry, Potentially toxic elements, Zn/Pb-bearing ore smelting, Human health hazard index, Pollution load index, Index of geoaccumulation

## Abstract

The main objective of the study was an assessment of the pollution level of agricultural land located close to dumps of industrial waste remaining after former Zn and Pb ore processing in Poland. The integrated geophysical-geochemical methods were applied for assessment of soil quality with respect to trace element pollution. Additionally, human health risk induced by the contaminated arable soil and dusting slag heap was estimated. The investigations pointed out that soils in the vicinity of the metallurgical slag dump in Piekary were heavily polluted. Spatial distribution of magnetic susceptibility corresponding well with distribution of the content of potentially toxic elements indicated the local “pollution hotspots.” Proper geophysical and geochemical data interpretation supported by statistical factor analysis enabled identification of three different sources of pollution including metallurgical slug dump as a main source, but also traffic pollution influencing the area located along the busy road and relatively strong influence of the geochemical background. Computed health hazard index revealed no adverse health effect to the farmers cultivating arable soil, but in the direct vicinity of dusting, slag dump health risk occurred, caused mostly by very toxic elements as As and Tl. In the future, investigation should be focused on contribution of different sources to the heavy metal pollution in soil-crop system in this area. It should be highlighted that a site-specific approach should be taken in order to redevelop this kind of area in order to reduce ecological and human health threat. The study proved the integrated two-stage geophysical-geochemical method to be a feasible, reliable, and cost-effective tool for identification of the extent of soil pollution and areas at risk.

## Introduction

Upper Silesia is the most urbanized and industrialized region of Poland and correspondingly the most polluted region of Poland (Dziubanek et al. 2017). Rich deposits of hard coal as well as zinc, lead, iron, and silver ores led to development of mining and metallurgical industry since the twelfth century. Nowadays, it is nearly impossible to find an area in this region that is not transformed by human activity. Numerous shafts, excavations, mine waste heaps, and subsiding troughs are inseparable views in Upper Silesia. There are numerous metal-rich waste heaps related to historical ore mining and processing being among the most significant sources of heavy metal contamination (Rożek et al. 2015; Warchulski et al. 2015). Such anthropogenic activities have generally influenced the conditions of water and soil (Merrington and Alloway 1997; Cabała et al. 2008). In addition, the presence of heavy metals may significantly influence the health of human beings as well as other organisms (Lanphear et al. 1998; Kozłowska et al. 2015). Hence, investigating and monitoring heavy metal pollution in soils is important. Soils in the vicinity and even at long distance from industrial sources are under influence of long-lasting emissions and deposition of potentially toxic elements (PTEs). These substances are present in urban and industrial dusts and can be deposited directly on soils or transported far away from the source (Steinnes et al. 1997; Steinnes and Friedland 2006).

Industrial emissions are also very important sources of technogenic magnetic particles (TMPs), mainly Fe oxides and hydroxides, which are generated in a wide variety of high-temperature technological processes, where different iron minerals, present in raw materials, fuels, and additives, are transformed into highly magnetic iron oxides (Magiera et al. 2011a). What is more, TMPs are known to be carriers of various pollutants such as heavy metals (Hunt et al. 1984; Beckwith et al. 1986; Strzyszcz 1993). Their presence in a material can be easily identified with magnetic susceptibility measurements, which are fast and relatively inexpensive. This proxy method based on the correlation between the topsoil magnetic susceptibility and the contents of PTE (mostly heavy metals) has been successfully used since the 1990s to detect and assess soil pollution with PTE (Strzyszcz et al. 1996; Petrovský et al. 2000; Wang and Qin 2006; Magiera et al. 2007; Jordanova et al. 2008; Cao et al. 2015). Therefore, magnetic susceptibility is an easy measurable general indicator of atmospherically derived particulate matter. The value of PTE itself reflects the general abundance of these elements, but to assess the overall level of contamination caused by particular or multiple elements, different methods and calculations, such as enrichment factor, contamination factor, geoaccumulation index (Igeo), pollution load index (PLI), and potential hazard index (HI), have been widely used (i.a. Jordanova et al. 2013; Li et al. 2014; Baran and Wieczorek 2015; Cao et al. 2015; Rachwał et al. 2015, 2017; Qing et al. 2015; Bourliva et al. 2017). Soil contamination by PTE, especially heavy metals and its possible consequences, was reported by many researchers from around the world where metal ore-bearing rocks have been exploited and smelted (McMartin et al. 2002; Svendsen et al. 2011; Šajn et al. 2013; Vaněk et al. 2013; Chrastný et al. 2015; Kapusta and Sobczyk 2015; Ettler 2016; Kríbek et al. 2016). Generally, the metallurgical wastes and ore processing itself were treated as the single pollution source.

The present study aimed at an assessment of the level of pollution of agricultural land located close to the dumps of industrial wastes remaining after Zn and Pb ore processing at Piekary Śląskie, located in the northern part of the Upper Silesian Industrial Region (USIR). This site was chosen among several pilot sites differing in character and extent of pollution which were investigated within the frame of the IMPACT project (development of integrated geophysical/geochemical methods of soil and groundwater pollution assessment and control in problematic areas) aimed at development of integrated two-stage geophysical/geochemical methods of evaluation of soil pollution and assessment of risk to sustainability of soil and groundwater. Moreover, an effort was made to assess the human health risk induced by the contaminated arable soil and dusting slag heap. This research applies magnetic susceptibility as the proxy tool, traditional geochemical analyses, computed pollution, and health hazard indices as well as correlation and PCA analyses. Therefore, it provides more complex, comparing with previous investigations, information about source-diversified soil contamination and its assessment in relation with human health.

## Studied area

The town of Piekary Śląskie (in the following named Piekary) is located in Silesia, southern Poland, near the city of Katowice. It covers an area of 40 km^2^ and has population of 54,600. The town is located at the Silesia-Cracow monocline in the region where the richest resources of zinc and lead ores in Poland occur. Strata-bound Zn-Pb deposits of the Mississippi Valley Type in this region consist mainly of sulfides occurring 40 to 240 m below surface. Those deposits are bounded to the Middle Triassic ore-bearing dolomites of Muschelkalk (Górecka 1993). Besides Pb and Zn, the deposits contain Cd; Fe; Mn; and in smaller amounts Ag, Sb, Cu, As, Tl, Ge, Co, Mo, Ni, Bi, Ba, and Sr (Gałkiewicz and Śliwiński 1983).

The Piekary area has been monitored during recent years for several parameters, e.g., Warchulski et al. (2015) reported a potential environmental impact of harmful elements (Pb, Zn, and As) leaching from smelting slags, while Ullrich et al. (1999) as well as Kulka and Gzyl (2008) concluded that soils in the Bytom and Piekary area were significantly contaminated by Zn, Pb, Cd, and As and should be withdrawn from agricultural production. Unfortunately, 60% of the study area consists of arable land where field workers cultivate consumable vegetables and where the main pollution source is not only the dusting slag heap but also industrial emissions, traffic-related pollution from the adjacent national road No. 94, and the contaminated soil itself. Therefore, the assessment of soil contamination level should be carried out parallel to simultaneous estimation of human health risk, which is caused mainly by metals and metalloids contained in investigated soils and wastes.

**Fig. 1:**
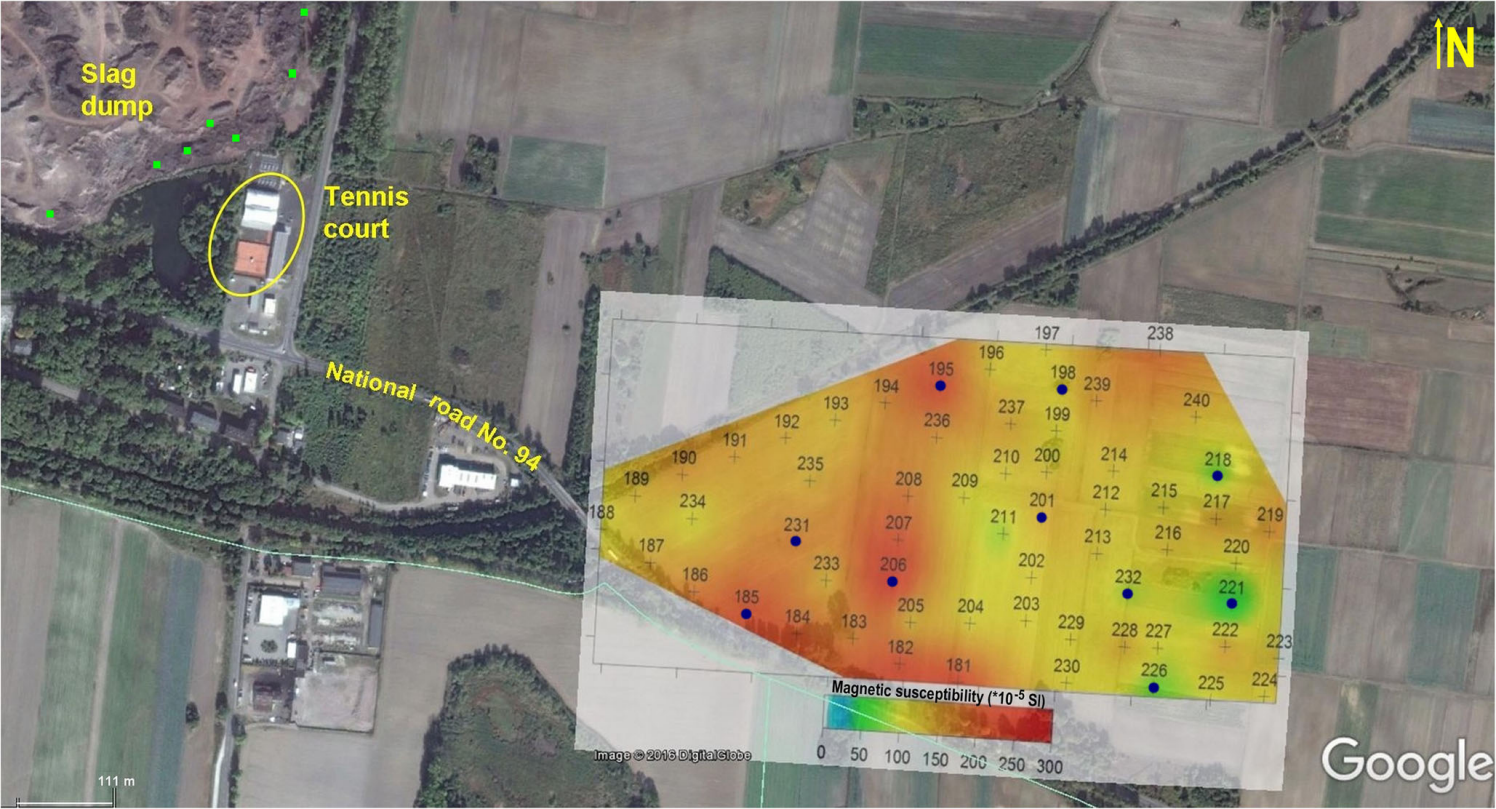
Study area and spatial distribution of к magnetic susceptibility (source of the base map: ©2016 Google Earth). Gray cross—measuring point. Blue dot—core sampling. Green square—slag sampling

Studies were carried out within a 1-km^2^ area of arable land. The field was located in the prevailing wind direction about 800 m east from a large dump of slag remaining after Zn and Pb ore processing (Fig. 1). Its topography is rather smooth with a gentle elevation (~ 10 m) in the eastern part of the field. These waste rocks originate from the former “Orzeł Biały” Mining and Smelting Company. The dump occupies about 16 ha and is about 20 m high. There is stored about 3.3 × 10^6^ m^3^ wastes (roll-down furnace slags with dolomite debris, agglomerate, and cinders of coke) with a mass of 5 million tons (Ferdyn and Strzyszcz 2002). Currently, the heap has been demolished in order to sell deposited dusts and slags, mainly for road building. For this purpose, a heavy earth-moving plant has been engaged, causing dusting of material rich in metals, especially Zn, Pb, and As, which content was estimated at the level of 0.26–5.9%, 0.03–4.0%, and 0.01–1.0%, respectively (Warchulski et al. 2015).

The soils in this area are rendzinas, developed on Triassic dolomites. They are fertile and were used for agricultural purposes for centuries. The main plants cultivated on this field are vegetables, such as beets, parsley, carrots, celery, etc. The south-western boundary of the studied field is a national road 94 from Zgorzelec (Polish boundary with Germany) to Korczowa (Polish boundary with Ukraine). According to a report of The General Directorate for National Roads and Motorways (GDDKiA 2017), average traffic density on this road is 15,088 vehicles per day (incl. 2346 of heavy duty trucks).

## Methods

### Magnetic investigations

The initial topsoil magnetic survey was performed using magnetic susceptibility meter MS2 Bartington equipped with MS2D loop sensor. At each individual point, the κ value was calculated as a mean value of 11 readings within a square of 2 m^2^. The κ value was expressed in 10^−5^ SI units. On this area, 60 points of surface magnetic susceptibility measurements were performed. Localization of individual points was confirmed by the Garmin GPS navigation device. Based on the κ measurements, a map of magnetic susceptibility distribution using Surfer 8 software (Golden Software Inc.) was prepared to select the most representative locations for soil sampling. Sampling was performed with application of Humax soil sampler enabling collection of undisturbed 30-cm soil cores. Two twin core samples (taken into plastic tubes at a distance <1 m apart) were taken in 10 points at different distances from the pollution source (0.75, 0.9, 1.0, and 1.1 km). Two additional cores were collected close to the road. Registrations of vertical distribution of volume magnetic susceptibility were made at intervals of 1 cm with application of MS2C Bartington sensor. The vertical distribution of magnetic susceptibility in twin topsoil cores did not always reveal identical shape, and the maximal concentration of TMPs (the layers of maximum κ values) was not at the similar depths; therefore, for further chemical analyses, samples from the uppermost (0–5 cm) and lower (5–15 cm) parts of arable soil as well as subsoil (below 15 cm) were collected. The cores were cut into three parts using a ceramic knife, and subsamples from relevant layers of the twin cores were mixed. A Bartington MS2B sensor was used for measurements of low field magnetic susceptibility (κ) of samples in order to calculate the mass-specific (χ) magnetic susceptibility by the following equation:

1 $$ \upchi =\mathrm{k}/\mathrm{q}\left({\mathrm{m}}^3{\mathrm{kg}}^{-1}\right), $$

where q is the density of material (Dearing 1994).

### Chemical analyses

Geochemical analyses were carried out at the Department of Chemistry of the Norwegian University of Science and Technology in Trondheim. For determination of PTEs, approximately 200–300 mg of dried soil sample was weighed into to a bottle containing 9 ml 50% *v*/*v* HNO_3_. After microwave digestion, each sample was diluted to 108 ml (109.8 ± 0.5 g) in Teflon bottle and then transferred to 15 ml PP-vials. Concentrations of elements were determined with High-Resolution Inductive Coupled Plasma-Mass Spectrometry (HR-ICP-MS) using an Element 2 instrument from Thermo Electronics. The main concern was on PTE that can migrate with aerosols and particulate matter as well as those expected in deposits (in alphabetical order): Ag, As, Ba, Cd, Cr, Cu, Fe, Ge, Hg, Mn, Ni, Pb, Se, Sr, Tl, V, and Zn.

Additionally, seven samples of materials from the neighboring slag heap were collected to check its influence on the chemical composition of soil from the studied field. Material collected from the heap was divided into two fraction: below 2 mm (fine fraction) and above 2 mm (coarse fraction). Magnetic susceptibility measurements were made in both fraction of samples and the corresponding χ values were calculated. Samples with fine fraction (below 2 mm) underwent the same chemical analysis as soil samples taken from the field.

Quality Assurance and Quality Control (QA/QC) was verified against reagent blanks, repeating test, and certified reference material (Soil GBW-07408). Concentrations in the reagent blanks were below detection limits for all elements. Accuracy of the employed procedure was checked by calculating the ratio between analyzed and certified values, and it was estimated at 60–82% of the certified value for almost all elements, and the confidence uncertainty was below 5% for As, Ba, Cu, Fe, Mn, Ni, Pb, Sr, Tl, V, and Zn; 5–15% for Ag, Cd, Cr, and Hg, while for Ge and Se, it was above 20%. All results were calculated back to solid material and corrected for blanks.

### Calculations and data analysis

The geoaccumulation index (Igeo) proposed by Müller (1969) defines the soil contamination by a given metal in terms of seven diversified classes. It is defined by the following equation:

2 $$ {\mathrm{I}}_{\mathrm{geo}}={\mathrm{log}}_2\left({\mathrm{C}}_{\mathrm{EL}}/1.5\ {\mathrm{C}}_{\mathrm{background}}\right), $$

where C_EL_ is the element content determined in soil samples, and C_background_ is the geochemical background concentration of the metal (Table 1). Factor 1.5 is the correction factor compensating the natural (lithological) fluctuations in the geochemical data. In this case, as the background values of all elements, the mean content calculated from the range of values commonly found in limestone and dolomites given by Kabata-Pendias and Pendias (2001) was applied in calculations of indices. Subsequently, the Tomlinson Pollution Load Index (PLI) was used to assess the overall contamination level of the soil (Tomlinson et al. 1980). Originally, the PLI was applied to assess the estuary pollution using the contents of heavy metals in the algae and flora as the indicators. At present, the PLI is often used to determine how much a set of contaminating elements in a given sample exceeds the background concentration. It also helps to compare the pollution level in places that differ in many aspects (e.g., localization in different climate zones, different industrialization levels, and land development). The PLI is defined as the *n*th root calculated from the product of *n* contamination factors (CF_EL_). To calculate the PLI, the following equations were used (Tomlinson et al. 1980):

3 $$ {\mathrm{C}\mathrm{F}}_{\mathrm{EL}}={\mathrm{C}}_{\mathrm{EL}}/{\mathrm{C}}_{\mathrm{background}} $$

4 $$ \mathrm{PLI}={\left({\mathrm{CF}}_{\mathrm{EL}1}\times {\mathrm{CF}}_{\mathrm{EL}2}\times {\mathrm{CF}}_{\mathrm{EL}3}\times \dots \dots \times {\mathrm{CF}}_{\mathrm{EL}\mathrm{n}}\right)}^{1/\mathrm{n}} $$

**Table 1:** Background values (Bkgrd) of elements (mgkg^−1^) commonly found in limestone and dolomites according to Kabata-Pendias and Pendias (2001) as well as contamination factors (CFs) of particular PTE (mgkg^−1^) and Igeo values given as arithmetic mean values of topsoil samples from the whole study area

PTE	Ag	As	Ba	Cd	Cr	Cu	Fe	Ge	Hg	Mn	Ni	Pb	Se	Sr	Tl	V	Zn
Bkgrd	0.13	1.7	125	0.35	10.5	6	7000	0.3	0.045	600	13.5	6.5	0.065	525	0.075	27.5	17.5
CF	4.0	10.7	1.8	36.7	3.1	3.7	2.2	3.4	5.0	1.0	1.2	69.9	8.0	0.1	13.3	1.3	59.3
Igeo	1.34	2.80	0.22	7.88	1.04	1.21	0.51	1.10	1.48	−0.53	−0.42	5.31	2.37	− 4.28	3.10	− 0.27	5.26

The CF_EL_ is the ratio of the content of each heavy metal (C_EL_) and its background value (C_background_, Tab. 1). EL1, EL2 … ELn are the contamination factors for individual pollutants. In the present case, almost all determined elements were involved in the PLI calculation; only elements with Igeo below 1.0 were excluded; therefore, *n* = 12 (Ag, As, Cd, Co, Cr, Cu, Ge, Hg, Pb, Se, Tl, and Zn).

For assessing the human non-cancer health risk posed by individual PTE contained in soil as well as material built the slag heap, a hazard quotient (HQ) was computed as the ratio of the average daily dose of chemical (ADD) to a reference dose (RfD) meaning the maximal tolerable daily intake of an element and established by USEPA (IRIS online database 2016). HQ values were calculated only for those elements with contamination factors exceeding 5.0 (Table 1), i.e., As, Cd, Hg, Pb, Se, Tl, and Zn. RfDs of these elements were established (Hough et al. 2004; USEPA 2014) as follows: 0.0003, 0.001, 0.0003, 0.035, 0.005, 0.00001, and 0.3 mg kg^−1^ day^−1^ for As, Cd, Hg, Pb, Se, Tl, and Zn, respectively:

5 $$ \mathrm{HQ}=\mathrm{ADD}/\mathrm{RfD} $$

Given that persons exposed to soil contaminants are mainly farmers cultivating investigated arable fields and workers rebuilding the slag heap, as well as tennis players from the heap-neighboring tennis court, the most important exposure pathways were considered to be soil ingestion (incidental consumption of soil on hands or food items), inhalation, and dermal absorption. Therefore, the total ADD of an element consists of totalized doses: from ingestion (ADD_I_), inhalation (ADD_Inh_), and dermal absorption (ADD_D_) which can be calculated according to the following equations (ATSDR 2004; Li et al. 2014; Qing et al. 2015):

6 $$ {\mathrm{ADD}}_{\mathrm{I}}=\left(\mathrm{C}\ast \mathrm{IR}\ast \mathrm{EF}\ast {\mathrm{ED}}^{\ast }{10}^{\hbox{-} 6}\right)/\left({\mathrm{BW}}^{\ast }\ \mathrm{AT}\right) $$

7 $$ {\mathrm{ADD}}_{\mathrm{Inh}}=\left({\mathrm{C}}^{\ast }\ {\mathrm{Inh}\mathrm{R}}^{\ast }\ {\mathrm{EF}}^{\ast }\ \mathrm{ED}\right)/\left({\mathrm{PEF}}^{\ast }{\mathrm{BW}}^{\ast }\ \mathrm{AT}\right) $$

8 $$ {\mathrm{ADD}}_{\mathrm{D}}=\left(\mathrm{C}\ast \mathrm{SA}\ast \mathrm{AF}\ast \mathrm{BF}\ast \mathrm{EF}\ast {\mathrm{ED}}^{\ast }{10}^{\hbox{-} 6}\right)/\left({\mathrm{BW}}^{\ast }\ \mathrm{AT}\right) $$

All symbols concerning hazard parameters used in Eqs. 7–9 are explained in Table 2. To assess the overall potential non-cancer health risk caused by multiple compounds, the hazard index (HI) was introduced as the sum of hazard quotients of individual elements (USEPA 1986). In the present work, HI values were calculated as follows:

9 $$ \mathrm{HI}=\Sigma\ \mathrm{HQ}={\mathrm{ADD}}_{\mathrm{As}}/{\mathrm{RfD}}_{\mathrm{As}}+{\mathrm{ADD}}_{\mathrm{Cd}}/{\mathrm{RfD}}_{\mathrm{Cd}}+{\mathrm{ADD}}_{\mathrm{Hg}}/{\mathrm{RfD}}_{\mathrm{Hg}}+{\mathrm{ADD}}_{\mathrm{Pb}}/{\mathrm{RfD}}_{\mathrm{Pb}}+{\mathrm{ADD}}_{\mathrm{Se}}/{\mathrm{RfD}}_{\mathrm{Se}}+{\mathrm{ADD}}_{\mathrm{Tl}}/{\mathrm{RfD}}_{\mathrm{Tl}}+{\mathrm{ADD}}_{\mathrm{Zn}}/{\mathrm{RfD}}_{\mathrm{Zn}} $$

**Table 2:** Hazard parameters used for calculating ADD_I_, ADD_I nh_, and ADD_D_

Symbol of hazard parameters	Explanation	Assumed value
C	Element content in soil	Specific for each element (mg kg^−1^)
IR	Ingestion rate^a^	100 mg day^−1^
Inhr	Inhalation rate of soil^c^	12.8 m^3^ day^−1^
EF	Exposure frequency^b^	66 days year^−1^
ED	Exposure duration	20 year
BW	Body weight^a^	70 kg
PEF	Particle emission factor^c^	1.36 × 10^9^ m^3^ kg^−1^
AT	The period over which the dose is averaged	365 days * ED
SA	Exposed skin surface area^b^	5700 cm^2^ (1/3 of the total skin surface area)
AF	Soil adherence factor^b^	0.07 mg cm^−2^ day^−1^
BF	Bioavailability factor^a^	0.1 (unitless)

^a^ATSDR 2004

^b^Kubicz 2014

^c^Qing et al. 2015

Hazard index values exceeding unity provides evidence that potential health effects may occur. Otherwise (HI < 1), it is assumed that the risk is at an acceptable level.

Moreover, the basic statistical functions of mean, median, standard deviation, Spearman correlation coefficients, and additionally factor analysis were applied (using Statistica 12 software; StatSoft) in order to analyze and interpret results and explain variations in the data.

## Results and discussion

### Magnetic properties

The preliminary field measurements show that above 80% of the studied area has κ values exceeding 100 × 10^−5^ SI units. The western part of the studied area, located closer to the waste dump, exhibits higher κ values (98–255 × 10^−5^SI units) than the eastern part (27–164 × 10^−5^SI units). The highest κ values are observed on the top of the local elevation in the central part of the studied area and along the road (Fig. 1). In addition, several magnetic hot spots related to the local morphology or diversity of soil composition are observed. Magnetic susceptibility map reflects a variation in soil composition and as a proxy for air-derived pollution—its level. There are any rules differentiating the level of magnetic signal responsible for a particular pollution level, but after three decades of application magnetic methods into environmental study, this approach becomes feasible and appropriate. Similar to the assessment of soil pollution caused by PTE, also in the case of magnetic susceptibility, some reference level should be considered. In the case of Piekary where rendzina soils occur, the background level is established as follows: 26.11 × 10^−5^ SI units and 42.16 × 10^−8^ m^3^ kg^−1^ for κ, as well as for *χ*, respectively (Jordanova 2016). Implementing these background values, the investigated area is characterized by magnetic susceptibility much higher than general background levels (κ _topsoil_ > 27.5 up to 255.5 × 10^−5^ SI; mean 139 × 10^−5^ SI and *χ* > 48 up to 295 × 10^−8^ m^3^ kg^−1^; mean 162 × 10^−8^ m^3^ kg^−1^) indicating soil contamination by TMPs.

**Fig. 2:**
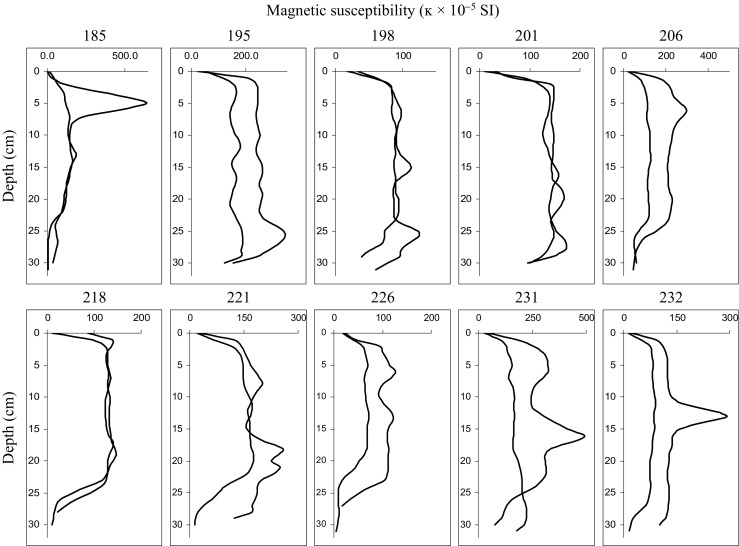
Vertical distribution of magnetic susceptibility (κ) in soil profiles from the study area (198, 201, 206, 218—typical distribution of arable soil profiles; 195, 221—distribution of arable soil profiles with some increase in ĸ in deeper layers; 185, 226, 231, 232—varied distribution of soil profiles with distinct peaks of κ values)

Vertical distribution of magnetic susceptibility in cores collected from sites 198, 201, 206, 218, and 226 is typical for arable land with homogenized upper soil layers due to plowing (Magiera et al. 2006, 2011b), i.e., in the upper 20 cm, the value of magnetic susceptibility is quite high (50–150 × 10^−5^SI units) and stable (Fig. 2), with a distinct drop below 20 cm. Similar trends are observed for magnetic susceptibility along cores from sites 221, 231, and 232 (Fig. 2), but with some deviations at 10–15 cm depth. These cores are characterized by increased κ values in the lower part, reaching values above 250 × 10^−5^SI units. In one core from site 185, a peak with very high (> 500 × 10^−5^SI units) magnetic susceptibility appears at a depth of 5 cm, probably resulted from accidental contamination during plowing. Even in points with the lowest surface values of κ (nos. 218 and 226), below 1–2 cm of depth, κ rises to a similar level observed at sites with higher magnetic susceptibility at the surface. Such high magnetic signal is not surprising since the *χ* of material collected from the slag heap ranged from 3031 to 5857 × 10^−8^ m^3^ kg^−1^ for fine, easily wind-blown fraction and from 2596 to 6930 × 10^−8^ m^3^ kg^−1^ for coarse one. The fine fraction of slag material was formed by physical weathering of initial material and was more homogenous than the corresponding coarse-grained fraction. However, the mean and median values of *χ* oscillate around 4000 × 10^−8^ m^3^ kg^−1^, which is a level principally characterizing industrial dusts and wastes (Magiera et al. 2011a).

### Geochemical properties

**Fig. 3:**
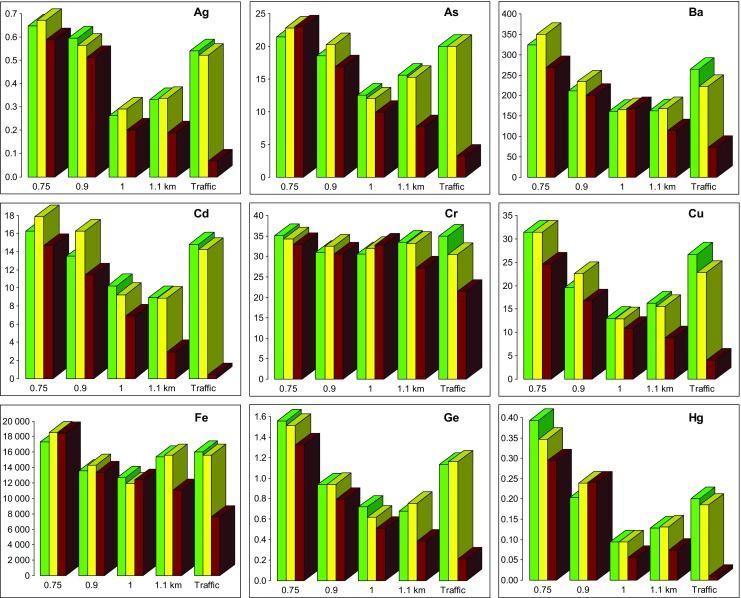

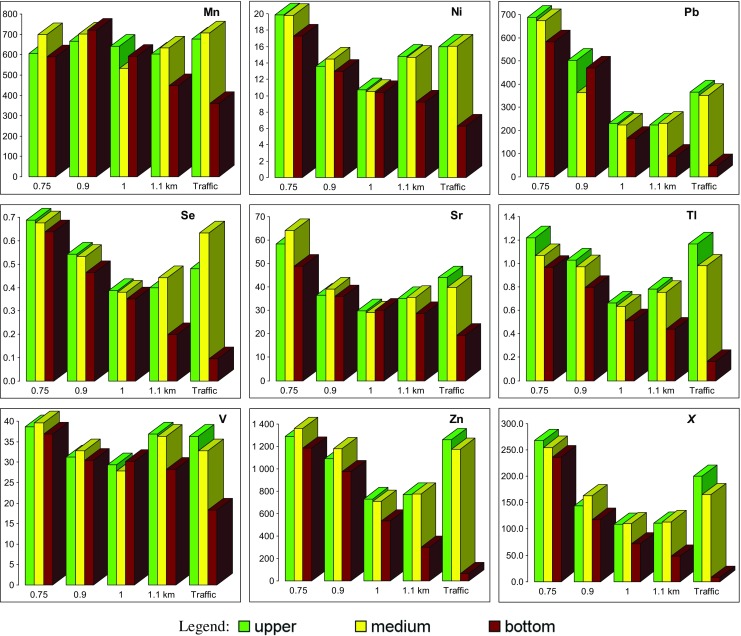
Distribution of PTE (in mg kg^−1^) presented in alphabetical order: Ag, As, Ba, Cd, Cr, Cu, Fe, Ge, Hg, Mn, Ni, Pb, Se, Sr, Tl, V, and Zn and the mass-specific magnetic susceptibility values (*χ* × 10^−8^ m^3^ kg^−1^) in different soil horizons (upper 0–5 cm; medium 5–15 cm; bottom below 15 cm) according to increasing distance from the metallurgical slag dump. The last bar represents samples collected close to the road at a distance of ~ 0.9 km

Soil in the studied area is characterized by neutral soil pH, which is typical for rendzinas, and high concentrations of almost all determined elements. The distributions of some elements correspond closely to the distribution of *χ* values (Fig. 3). The highest amounts of Ag (> 0.6 mg·kg^−1^), As (> 20 mg·kg^−1^), Ba (> 250 mg·kg^−1^), Cd (> 15 mg·kg^−1^), Cu (> 30 mg·kg^−1^), Ge (> 1.5 mg·kg^−1^), Hg (> 0.3 mg·kg^−1^), Ni (> 18 mg·kg^−1^), Pb (> 600 mg·kg^−1^), Se (> 0.6 mg·kg^−1^), Sr (> 50 mg·kg^−1^), Tl (> 1 mg·kg^−1^), and Zn (> 1200 mg·kg^−1^) were observed in the western part of the studied area at a distance of 0.75 km from the source. Chromium and manganese show a stable high concentration through the whole study area, and the content of all the other elements decreases with distance from slag heap—up to 1 km and then start to increase again in the most elevated sites lying most distant from the source (1.1 km distance). The last column on the bar chart (Fig. 3) represents cores sampled close to the road and therefore being influenced mostly by traffic-related emissions, although the impact of secondary emission from a slag dump (about 0.9 km away from that site) cannot be neglected. It is characterized by enhanced values of *χ* as well as contents of Ag, As, Ba, Cd, Cu, Ge, Hg, Ni, Pb, Se, Tl, and Zn.

According to the limit values proposed by regulations from Swiss Agency for the Environment, Forests, and Landscape, based on nitric acid extraction (Desaules et al. 2001), contents of Zn, Pb, and Cd in all investigated samples (except subsoil at site no. 185) exceed threshold values for arable soils (150; 50 and 0.8 mg kg^−1^, respectively). The contents of barium and arsenic are also relatively high, but these elements are not considered in the above regulations. Similar to the magnetic results, an effect of plowing is observed in the PTE content. Level of elements is similar at different depths (0–5 and 5–15 cm) due to mixing of soil horizons during agricultural treatment. Only in case of profiles lying farther from the slag dump (1–1.1 km), as well as soil profile influenced by traffic, PTE content in the deeper layers is definitely lower. Contents of Cr, Fe, Mn, and V are relatively evenly distributed, independent of the distance from the slag dump as well as the soil horizon. Such a distribution can be explained not only by agriculture treatment but also by geological background and leaching processes downwards the soil profile (Degryse and Smolders 2006). In general, however, enhanced concentrations of most PTE are observed at 0–15 cm depth, decreasing toward the bottom end. This may point at the influence of anthropogenic activity in this region and especially re-suspension from adjacent smelting slag heap characterized by very high values of magnetic susceptibility (*χ* above 2500 × 10^−8^ m^3^ kg^−1^). The rich chemical composition of dumped wastes is strongly dominated by Fe, Zn, Pb, As, Mn, and Cu; Ba, Ni, Cr, Cd, and Tl have enhanced contents as well, while the content of Se is below detection limit (Table 3).

**Table 3:** Statistics concerning content of individual PTE (mgkg^−1^) as well as mass-specific magnetic susceptibility (10^−8^ m^3^ kg^−1^) in the material collected from smelting slag heap (Se—below detection limit)

	As	Ba	Cd	Cr	Cu	Fe	Mn	Ni	Pb	Tl	Zn	*χ*
Min	1579	324	3	27	2877	44,800	2320	123	2968	0	8483	3031
Max	9692	655	101	59	8784	119,400	10,727	307	9456	129	37,083	5857
**Ave.**	**5264**	**531**	**26**	**45**	**4752**	**80,400**	**4935**	**186**	**6144**	**26**	**15,476**	**4177**
**Med.**	**4720**	**567**	**16**	**45**	**3618**	**81,100**	**3684**	**170**	**7072**	**26**	**12,909**	**4085**
St. Dev.	2446	128	32	9	2272	23,700	2765	60	2382	39	9075	952

Bold entries indicate only the lines with mean and median values

### Soil pollution source apportionment

Results of factor analysis confirm two different sources of element load in the topsoil and subsoil as well (Table 4). The first factor (explaining 70 and 83% of the variance for topsoil and subsoil, respectively) combines magnetic susceptibility and PLI as well as most individual elements (Ag, As, Ba, Cd, Co, Cu, Fe, Ge, Hg, Ni, Se, Sr, Tl, and Zn), i.e., variables mainly connected with the composition of the slag heap. The second factor (16 and 10% of the variance for topsoil and subsoil, respectively) comprises mainly Cr, Pb, and V, which may all be connected with traffic-related emissions: Cr can originate from brake linings, engine oils, fuel, and road abrasion (Wiseman et al. 2015; Wang et al. 2017), while Pb and V (as well as Ni and Cd)—from pipe emissions and heavy fuel oil combustion of both gasoline and diesel engines (Wilhelm et al. 2012; Çolak et al. 2016). Additional important source of Pb was wear abrasion or also earlier depositions (as Pb was present in petrol in Poland until 2005) (Wawer et al. 2017). Moreover, taking into consideration that the investigated area is intensively utilized for agricultural production, the fields were most likely fertilized in order to yield heavier crops. As a consequence, continued long-term fertilization can increase soil metal contents or their bioavailability, even when these occur at low concentrations (Leita et al. 1999).

**Table 4:** Results of factor analysis of the data variability for topsoil (0–5 cm) and subsoil (below 15 cm)

Element	Topsoil		Subsoil	
	F1	F2	F1	F2
Ag	− 0.79		− 0.92	
As	− 0.92		− 0.98	
Ba	− 0.97		− 0.99	
Cd	− 0.85		− 0.92	
Co	− 0.87		− 0.88	
Cr		0.75	− 0.75	0.58
Cu	− 0.99		− 0.997	
Fe	− 0.77		− 0.84	
Ge	− 0.96		− 0.97	
Hg	− 0.97		− 0.88	
Mn			− 0.64	
Ni	− 0.88		− 0.95	
Pb		− 0.75	− 0.74	− 0.60
Se	− 0.93		− 0.98	
Sr	− 0.94		− 0.97	
Tl	− 0.94		− 0.97	
V		0.74	− 0.82	0.54
Zn	− 0.93		− 0.96	
MS	− 0.95		− 0.96	
PLI	− 0.84		− 0.98	
Variance explained (%)	70	16	83	10

*MS* mass-specific magnetic susceptibility, *PLI* pollution load index

As enhanced magnetic susceptibility indicated soil contamination caused by air-derived particulate matter, the significant relationships between magnetic susceptibility (related to the amount of TMPs being the carriers of heavy metals) and content of particular PTE can be considered as an indicator pointed at a similar source of pollution (Zawadzki et al. 2015; Lu et al. 2016; Bourliva et al. 2017). In this study, significant and relatively high correlation coefficients (0.61–0.97, Table 5) were observed for topsoil in the case of Ag, As, Ba, Cd, Co, Cu, Fe, Ge, Hg, Ni, Se, Sr, Tl, and Zn, which all may be related to past and present pollution sources. A similar relationship is noticed for PLI as well as between PLI and the same, abovementioned PTE, suggesting re-suspension from the adjacent metallurgical slag dump as the main source of elevated contents of PTE and enhanced *χ*. Obviously, the nearby busy road and several small enterprises may also have contaminated the studied soils. Undoubtedly, the lithology of this area (ore-bearing dolomites) has a significant effect on its geochemistry. Negative results of Igeo demonstrate background concentrations; therefore, Mn, Ni, Sr, and V, as well as Ba (with Igeo ~ 0.2), can be considered as elements originated from geological background.

**Table 5:** Correlation matrix of investigated elements for topsoil (*n* = 12; correlation coefficients in bold are significant with *p* < 0.05)

	Ag																		
As	**0.84**	As																	
Ba	**0.74**	**0.84**	Ba																
Cd	**0.85**	**0.91**	**0.82**	Cd															
Co	0.43	**0.67**	**0.88**	**0.62**	Co														
Cr	0.15	0.41	0.55	0.19	**0.76**	Cr													
Cu	**0.77**	**0.88**	**0.97**	**0.83**	**0.85**	0.56	Cu												
Fe	0.31	**0.64**	**0.72**	0.39	**0.86**	**0.90**	**0.75**	Fe											
Ge	**0.72**	**0.80**	**0.98**	**0.83**	**0.90**	0.55	**0.96**	**0.69**	Ge										
Hg	**0.80**	**0.87**	**0.93**	**0.85**	**0.86**	0.51	**0.95**	**0.70**	**0.95**	Hg									
Mn	0.16	0.38	− 0.05	0.38	− 0.09	− 0.15	0.01	− 0.07	− 0.09	0.01	Mn								
Ni	0.51	**0.72**	**0.86**	0.52	**0.91**	**0.82**	**0.87**	**0.95**	**0.84**	**0.84**	− 0.19	Ni							
Pb	**0.65**	0.30	0.24	0.41	− 0.10	− 0.39	0.31	− 0.22	0.22	0.34	0.04	− 0.01	Pb						
Se	**0.80**	**0.92**	**0.90**	**0.88**	**0.80**	0.41	**0.87**	**0.63**	**0.87**	**0.92**	0.26	**0.75**	0.28	Se					
Sr	**0.69**	**0.82**	**0.89**	**0.74**	**0.82**	0.55	**0.95**	**0.76**	**0.89**	**0.92**	0.04	**0.85**	0.40	**0.82**	Sr				
Tl	**0.87**	**0.93**	**0.90**	**0.89**	**0.72**	0.47	**0.91**	**0.64**	**0.88**	**0.91**	0.11	**0.76**	0.28	**0.88**	**0.79**	Tl			
V	0.17	0.47	0.56	0.18	**0.76**	**0.96**	0.58	**0.96**	0.53	0.55	− 0.13	**0.88**	− 0.33	0.46	**0.61**	0.49	V		
Zn	**0.88**	**0.95**	**0.90**	**0.95**	**0.69**	0.40	**0.92**	0.56	**0.88**	**0.88**	0.28	**0.68**	0.35	**0.89**	**0.82**	**0.95**	0.39	Zn	
*χ*	**0.76**	**0.84**	**0.95**	**0.88**	**0.81**	0.37	**0.97**	**0.61**	**0.96**	**0.94**	0.00	**0.77**	0.39	**0.87**	**0.91**	**0.87**	0.41	**0.89**	χ
PLI	**0.74**	**0.77**	**0.85**	**0.77**	**0.65**	0.25	**0.85**	0.50	**0.83**	**0.80**	− 0.01	**0.66**	0.43	**0.78**	**0.82**	**0.76**	0.30	**0.82**	**0.87**

### Assessment of soil contamination

**Fig. 4:**
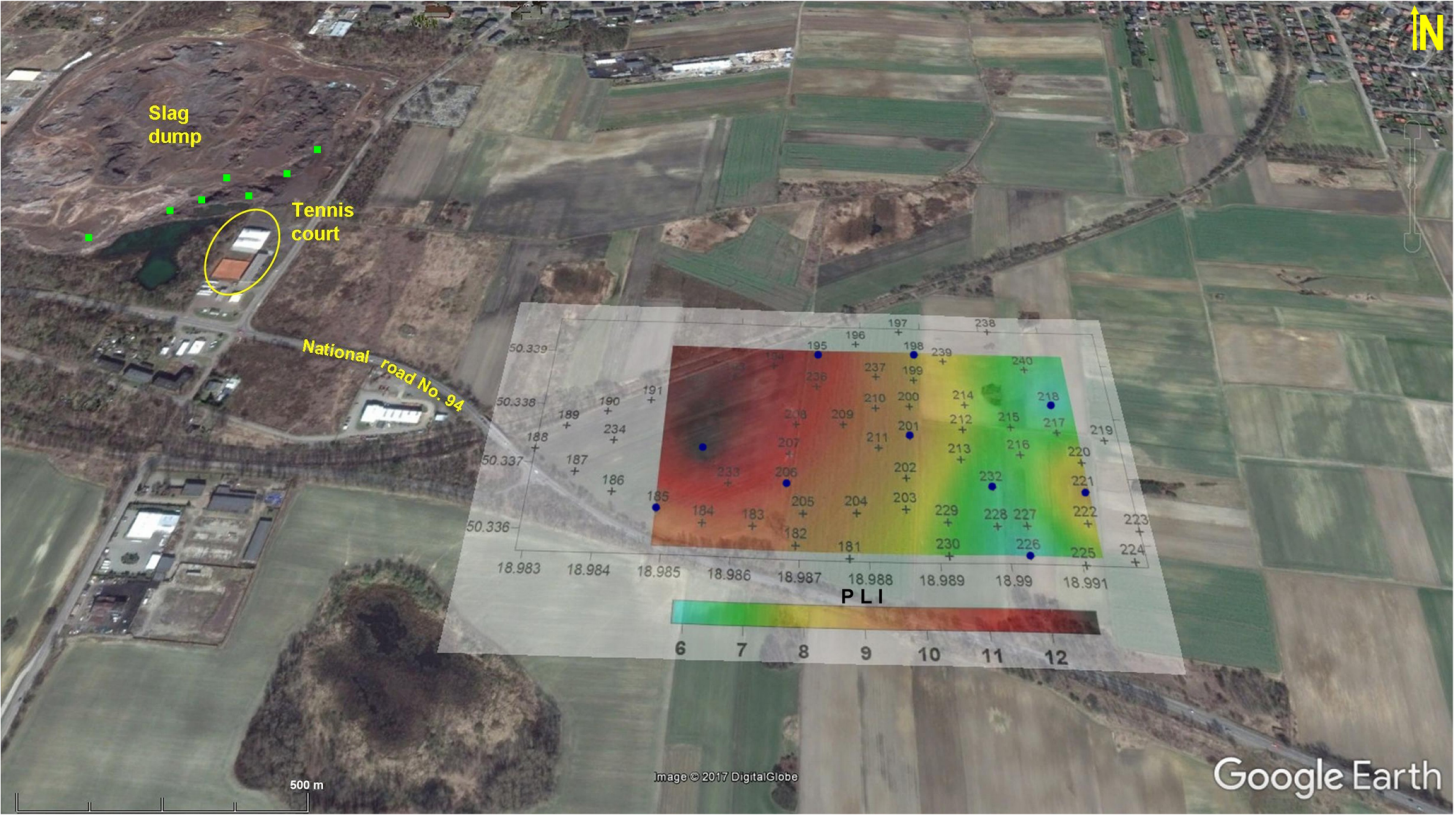
The spatial distribution of PLI in the topsoil (0–5 cm) of the area of study (source of the base map: ©2016 Google Earth); gray cross—measuring point, blue dot—core sampling, green square—slag sampling

**Fig. 5:**
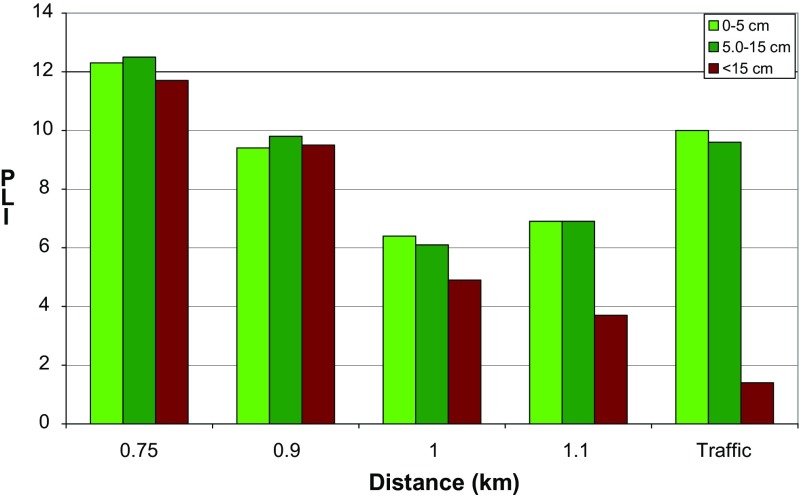
PLI values of soil (from different depths 0–5, 5–15, and < 15 cm) from the study sites ordered according to the increasing distance from metallurgical slag dump calculated on the basis of 11 elements. The last column represents samples collected close to the road

While assessing soil contamination, the results should be compared with the so-called reference level which can be understood as a background value or some acceptable threshold value above which an environmental or human health hazard can occur (Reimann and de Caritat 2005; Gałuszka 2007; Desaules 2012). Such a comparative method is the PLI, often used for more complex comparison between different geographic sites (e.g., Rachwał et al. 2015), but in the present case, its spatial distribution reflects the extent of contamination in a better way than contents of individual elements. The highest PLI values occur in the north-western part of the field, decreasing in the south-east direction; hence, the PLI map indicates unequivocally the re-suspension from the adjacent smelting slag dump as the main source of soil contamination by PTE (Fig. 4). Nevertheless, the soil magnetic susceptibility distribution signifies an additional source of contamination in the southern part of the field, next to national road no. 94 (Figs. 4 and 5). According to PLI classification (Tomlinson et al. 1980), the obtained results describe the topsoil of the whole area as extremely heavily polluted (PLI > 3), although with a distinct difference between the more distant eastern (6.4 < PLI < 9.3) and the closer to slag dump western part of the area exhibiting higher level of pollution (9.4 < PLI < 12.3) (Fig. 4). The PLI decreases slightly with the depth, especially at most distant sites. In the case of the sampling points located closer to the dump, increase in PLI at the medium level of soil profiles (5–15 cm) is stated. It can be explained by agrotechnical treatments as well as by leaching of some elements into deeper soil horizons.

Diversified results of Igeo reveal that in the Piekary area, the entire soil profiles are extremely contaminated by Cd, Zn, and Pb (4.5 < mean Igeo < 8) followed by As, Se, and Tl (2 < mean Igeo < 3.2) which, according to Müller’s classification (1969), exhibit the third Igeo class, i.e., moderately to strongly contaminated (Table 1). In contrast, Ba, Fe, Mn, Ni, Sr, and V are characterized by very low values of Igeo in all soil horizons suggesting no contamination, or in case of Ba and Fe (0.3 < mean Igeo < 0.56) uncontaminated or moderately contaminated soil (Müller 1969).

### Assessment of human health risk

Although the study area is known for the significant contamination caused by non-ferrous metallurgy as well as dumping of the smelting slags and processing wastes (Rybicka 1996; Ullrich et al. 1999; Kulka and Gzyl 2008), the fields are kept cultivated for food production (e.g., cabbage, onion, cauliflower), regardless of the possible health risk posed by consumption of local vegetables. Heavy metals, such as Cd, Hg, Cr, Pb, and As, can be easily transferred from soil to leaf vegetables (Liu et al. 2013; Chang et al. 2014). The pollutants can enter human bodies through three pathways: oral ingestion, dermal contact, and inhalation of soil and dust particles.

The potential human non-cancer health risk is assessed by means of HQ and HI (Table 6). The order of HQ for particular elements is as follows: Tl > As > Pb = Cd > Zn > Hg > Se for the cultivated field and As > Tl > Pb > Zn > Cd for slag heap. For the field, the HI does not exceed unity; therefore, deleterious effect for farmers should not occur.

**Table 6:** HQs of individual heavy metals and HIs for the area of study

	Whole area	Site no. 195	Slag heap
As	0.22	0.26	63.41
Cd	0.05	0.06	0.09
Hg	0.0027	0.0038	–
Pb	0.05	0.12	0.63
Se	0.0004	0.0005	–
Tl	0.36	0.38	9.36
Zn	0.01	0.01	0.19
**HI**	**0.693**	**0.834**	**73.68**

Bold entries indicate only the lines with HI values as a target of calculations

Surprisingly, our research estimates the non-cancer hazard index at a level below unity, demonstrating no adverse health effect to the residents, especially those working in the arable field. It should be pointed out that in HI, computing the dietary intake was not taken into consideration; therefore, the health risk for field workers will increase due to consumption of crops. Unfortunately, the industrial workers rebuilding the slag dump are seriously endangered by PTE occurring in the dumped material because the HI is extremely high and equals almost 74, the risk is enhanced by the fact that As and Tl, both very toxic elements, are the main contributors for this value. Moreover, right at the foot of the dump, a tennis court is located, posing some potential health risk for the tennis players, especially in windy weather. Still, comparing the present data with other similar studies, the overall health threat appears to be quite low. For example, Bourliva et al. (2017) stated that in an industrial area near Thessaloniki hazard quotient contributed by Pb alone exceeded 10. Similarly, in the most important mining and metallurgical centers in Spain, HI amounted to 9.3 with arsenic as the main component (Wcisło et al. 2016). In the Piekary area, the highest contribution to HI was provided by As together with Tl, both possessing highly toxic and carcinogenic properties (Wierzbicka et al. 2004; Rieuwerts et al. 2006; Núñez et al. 2016).

## Conclusions

The results reported show that an integrated two-stage geophysical/geochemical method provides straightforward estimates of soil pollution, which together with application of pollution and health hazard indices can be used for assessment of soil contamination and the human health risk of PTE transported by the air from the local source of pollution and deposited on the cultivated soil layer. The investigations pointed out that soils in the vicinity of the metallurgical slag dump in Piekary are heavily polluted. Spatial distribution of magnetic susceptibility pointed at local “pollution hotspots,” while PLI reflected the general trend in distribution of PTE. Proper geophysical and geochemical data interpretation supported by statistical principal component analysis enabled identification of at least three different sources of pollution including metallurgical slug dump as a main source, but also traffic pollution influencing the area located along a busy road and relatively strong influence from the geochemical background. The latter source was related to the layer of weathered dolomites present in the background rock, which enriched the subsoil in elements such as Ba, Mn, Ni, Sr, and V. In spite of the relatively high geochemical background, the correlations in topsoil between magnetic susceptibility and contents of anthropogenically derived PTE were high (> 0.8) for such elements such as As, Ba, Cd, Co, Cu, Ge, Hg, Se, Sr, Tl, and Zn. In the light of present results concerning soil contamination, long lasting and regularly farming of these fields may pose a real threat to human health. Therefore, health hazard index was computed, but it revealed no adverse health effect to the farmers cultivating arable soil (HI below 1). However, the high health risk (HI ~ 74) occurred in the direct vicinity of dusting slag dump, caused by very toxic elements as As and Tl. Unquestionably, the investigated area is highly polluted and may pose a human health threat. Therefore, regardless of the present results, more detailed site surveys (involving air, soil, water, and consumable plants and crops) should always be required when assessing a specific site within an urban area with agriculture land use. Further investigations should be focused on contribution of different sources to the heavy metal pollution in soil–crop systems in this area. A site-specific approach should be used to redevelop this kind of area in order to reduce ecological and human health hazard.
